# Eavesdropping on Plant Volatiles by a Specialist Moth: Significance of Ratio and Concentration

**DOI:** 10.1371/journal.pone.0017033

**Published:** 2011-02-09

**Authors:** Dong H. Cha, Charles E. Linn, Peter E. A. Teal, Aijun Zhang, Wendell L. Roelofs, Gregory M. Loeb

**Affiliations:** 1 Department of Entomology, New York State Agricultural Experiment Station, Cornell University, Geneva, New York, United States of America; 2 Chemistry Research Unit, Center for Medical, Agricultural and Veterinary Entomology, Agricultural Research Service, United States Department of Agriculture, Gainesville, Florida, United States of America; 3 Invasive Insect Biocontrol and Behavior Laboratory, Plant Sciences Institute, Agricultural Research Service, United States Department of Agriculture, Beltsville, Maryland, United States of America; AgroParisTech, France

## Abstract

We investigated the role that the ratio and concentration of ubiquitous plant volatiles play in providing host specificity for the diet specialist grape berry moth *Paralobesia viteana* (Clemens) in the process of locating its primary host plant *Vitis* sp. In the first flight tunnel experiment, using a previously identified attractive blend with seven common but essential components (“optimized blend”), we found that doubling the amount of six compounds singly [(*E*)- & (*Z*)-linalool oxides, nonanal, decanal, β-caryophyllene, or germacrene-D], while keeping the concentration of other compounds constant, significantly reduced female attraction (average 76% full and 59% partial upwind flight reduction) to the synthetic blends. However, doubling (*E*)-4,8-dimethyl 1,3,7-nonatriene had no effect on female response. In the second experiment, we manipulated the volatile profile more naturally by exposing clonal grapevines to Japanese beetle feeding. In the flight tunnel, foliar damage significantly reduced female landing on grape shoots by 72% and full upwind flight by 24%. The reduction was associated with two changes: (1) more than a two-fold increase in total amount of the seven essential volatile compounds, and (2) changes in their relative ratios. Compared to the optimized blend, synthetic blends mimicking the volatile ratio emitted by damaged grapevines resulted in an average of 87% and 32% reduction in full and partial upwind orientation, respectively, and the level of reduction was similar at both high and low doses. Taken together, these results demonstrate that the specificity of a ubiquitous volatile blend is determined, in part, by the ratio of key volatile compounds for this diet specialist. However, *P. viteana* was also able to accommodate significant variation in the ratio of some compounds as well as the concentration of the overall mixture. Such plasticity may be critical for phytophagous insects to successfully eavesdrop on variable host plant volatile signals.

## Introduction

Understanding the mechanisms used by herbivorous insects to recognize and find suitable hosts has ecological, evolutionary and economic significance as herbivorous insects play vital roles in ecosystem functioning by mediating transfer of energy and nutrients [1], while also competing with humans for food [2]. Volatiles play a critical role in the evolution of host use by phytophagous arthropods [3] and the ability to efficiently eavesdrop on the host plant from a distance [4], [5] has contributed to the success of these organisms [6]. Contrary to initial expectations, the majority of phytophagous insects use a mixture of ubiquitous volatile compounds, rather than unique, host-specific compounds in the host finding process [7], [8]. Complicating matters further, volatile emissions from plants are not static but greatly vary due to a number of biotic and abiotic factors [9]–[12]. This raises the question of how phytophagous insects extract host specific information from the mixture of common plant volatiles that vary in both time and space.

Although release of plant volatiles occurs, to some extent, as the unintended consequence of biochemical processes [13], there is good evidence that plant volatiles serve important plant fitness enhancing functions, such as pollinator attraction or natural enemy recruitment [14]–[19]. In contrast, phytophagous insects are not the intended receiver of the host volatiles, and may have been selected for the ability to eavesdrop on the volatiles for their own advantage, whether to colonize a suitable host or avoid an unsuitable one [20], [21].

A mechanistic understanding of how variable plant volatile signals mediate exploitative interactions between plants and herbivores is limited [21], [22], largely due to the lack of information on the behaviorally relevant key essential volatiles used by specific herbivorous insect species [12], [23]. A similar challenge exists for understanding other multi-trophic, plant fitness enhancing interactions, for example with pollinators and natural enemies [16], [20], [22], [24]–[26]. Gas chromatography–electroantennographic detection (GC-EAD) techniques have shown that the chemoreceptors on the antennae of any particular species can only detect a portion of the released volatiles and often the compounds that dominate the volatile blend are not necessarily the most important compounds in terms of behavior [23], [27]–[32]. Thus, to extract information from a relatively limited number of combinations of ubiquitous volatiles, insect herbivores must rely on specific features of the signal, such as composition, ratio or concentration [7], [33], [34]. There are few empirical data that validate these predictions [8], [12], although recent studies have begun to show how behaviorally relevant odors, such as flower volatiles used by pollinators, are encoded in the antennal lobe of the insect brain [31], [32]. It remains unclear how insects recognize ratios or concentrations of volatile blends [34]. Therefore, a main objective of the research reported here was to understand to what extent specificity of a common volatile mixture depends on ratio and concentration for a specialist herbivorous insect.

Grape berry moth, *Paralobesia viteana* (Clemens) (Lepidoptera:Tortricidae) is an excellent study organism to evaluate the effect of changes in ratio or concentration on the specificity of volatile blends because the volatile cues and behavioral system are well understood. For example, in previous studies using GC-EAD and flight-tunnel bioassays, an essential volatile blend was identified [23] that (1) was as attractive to female *P. viteana* as their host plant in a specific ratio and concentration, (2) lost attractiveness completely when any of the compounds were removed from the mixture, and (3) successfully captured *P. viteana* when used as a lure in traps under field conditions. In addition, as a diet specialist that is expected to be more efficient in locating host plants compared to generalists [6], *P. viteana* is a good candidate to test the effect of blend quality on the variation in response specificity.

Here we present the results of flight tunnel studies on the effect of ratio and concentration on the specificity of an essential volatile blend in *P. viteana*. We focused on a previously identified attractive 7 component blend for GBM [23] and manipulated ratio and concentration of the blend by systematically doubling single compounds or by mimicking changes in volatile emission caused by Japanese beetle feeding on grapevines [35]. Our results support the hypothesis that *P. viteana* is responsive to changes in the ratios of some, but not all, compounds in a blend. The results also show that a relatively small modulation in ratio of volatile constituents was responsible for the decrease in attractiveness of grape shoots damaged by Japanese beetle feeding, although increased concentration as a result of beetle feeding damage did not reduce female response within the concentration range we tested.

## Methods

### Insects

*P. viteana* were reared as previously described in walk-in environmental chambers at 26°C and 60% RH under a 16:8 (L:D) photoperiod [23]. Adult moths were mated freely in rearing cages and oviposited on grapes. Four first instar larvae were transferred to a 30 ml diet cup and reared on semi-synthetic diet. To minimize any potential effect of laboratory rearing on the behavior of moths, colonies were re-established every year using field-collected larvae.

### Experiment 1: *P. viteana* response to artificial manipulation of ratio of key volatiles

#### Synthetic blends—doubling single compounds

We used a previously identified 7-component blend composed of a racemic mixture of (*E*)- & (*Z*)-linalool oxides, nonanal, decanal, 4,8-dimethyl-1,3(*E*),7-nonatriene (tDMNT), β-caryophyllene and germacrene-D as the “optimized blend” (prepared at 0.1 µg total compounds/µl hexane) mixed according to the ratio provided in Table 1, which was optimized for maximal upwind orientation in the flight tunnel [23]. To test for the effect of ratio, we modified the optimized blend by doubling the amount of each component singly, while holding the amount of the remaining compounds constant. Doubling the concentration of each volatile was done to test the hypothesis that this specialist insect should be sensitive to a particular volatile blend. Thus, if ratio is critical, we would expect a significant effect on female behavior with the relatively small increase in doubling the concentration.

**Table 1:** The relative ratios (%) of the seven key essential volatile compounds used in three synthetic blends.

Essential compounds	Optimized blend (%)	JB undamaged blend (%)	JB damaged blend (%)
(E)- & (Z)-Linalool oxides	13	1	2
Nonanal	13	2	0.2
Decanal	13	3	0.8
(*E*)-4,8-dimethyl 1,3,7-nonatriene	30	53	70
β-caryophyllene	7	18	12
Germacrene-D	24	23	15

Optimized blend was previously identified and optimized for flight tunnel activity of female *P. viteana* [23]. Ratio of Japanese beetle (JB) damaged and undamaged blends were based on GC/MS analyses of volatiles collected from Japanese beetle damaged and undamaged *V. riparia* shoots, respectively.

#### Chemicals

All synthetic compounds, except tDMNT and germacrene-D, were greater than 97% in purity and obtained from Sigma-Aldrich (St. Louis, MO, USA), Alfa Aesar (Ward Hill, MA, USA), Fluka (Buchs, Switzerland) or TCI America (Portland, OR, USA). The tDMNT was synthesized as mixture of 95% (*E*)-isomer and 5% (*Z*)-isomer. Germacrene-D was isolated from golden rod (*Solidago* species) as 91% germacrene-D and 9% β-caryophyllene (see [23] for more detail).

#### Flight Tunnel Assays

Flight-tunnel assays were performed using five-day-old mated female *P. viteana* as described previously [23]. The flight tunnel (2 m ×0.6 m ×0.6 m) conditions were as follows: wind speed at 0.25 m/sec, light intensity at 25 lux, temperature at 23.5°C (±2.0 *SD*), and relative humidity at 53.2% (±9.2 *SD*). Tests were conducted during the first 2 hours of the scotophase period. An individual moth was released from a metal screen cage located 1.5 m downwind of the target, a rubber septum (11 mm OD, Kimble Kontes, Vineland, NJ, USA) loaded with 300 µl (30 µg) of the synthetic blend, placed at the upwind end in the center of tunnel. We scored each moths for specific behaviors: (1) left cage (when they left the release cage), (2) partial upwind flight (when they made more than 50 cm of zig-zag upwind flight, a similar behavior as observed in male *P. viteana* flights to a sex pheromone lure, up to 10 cm of the target), (3) full upwind flight (upwind flight within 10 cm of the target), or (4) landing (contact with target).

### Experiment 2: *P. viteana* response to Japanese beetle damage-induced modulation of ratio and concentration of key volatiles

In Experiment 1, doubling a single compound resulted in 7–30% increase in concentration of the blend, depending on which compound was doubled (Table 1). For example, doubling nonanal (13%) in the optimized blend of 0.1 µg/µl would result in 0.113 µg/µl concentration of the nonanal doubled blend—a 13% increase in total concentration. To help decouple the influence of ratio and concentration on female behavior, in Experiment 2 we modified ratio and concentration independently based on changes caused by Japanese beetle damage.

#### Plants and Insects

We used a native host species of *P. viteana* in the northeastern and central U. S., *Vitis riparia*. Cuttings from a single clone were made in December 2008, rooted and transplanted to 1 gallon pots, and grown in a greenhouse under supplemental light (16 h day length) and weekly fertilization. A total of eight clonal plants were used for this experiment. *Popillia japonica* (Japanese beetles) were collected from *V. riparia* vines growing naturally in the vicinity of Geneva, NY (August 3–12, 2009) and allowed to feed on half of the clonal vines. Four vines damaged by adult Japanese beetles were subjected to headspace volatile collection and flight tunnel bioassays. We also collected volatiles from four remaining undamaged vines and tested the undamaged vines in the flight tunnel. To create damage, a single potted grape vine was enclosed in a screened chamber (45 cm H ×77 cm W ×45 cm D) in the green house with 40 field collected Japanese beetles over night. Japanese beetles removed an average of 10–20% leaf area estimated by visual inspection [36]. After treatment, Japanese beetles were released unharmed.

#### Adsorbent Sampling

We collected the headspace volatiles from live shoots of potted grape vines using a push-pull collection glass chamber as previously described [23]. For each grapevine, two 60 cm grape shoots were carefully placed in the chamber. We pushed filtered air into the chamber at 1.0 l/min (ARS Inc., Gainesville, FL, USA) and collected shoot volatiles onto charcoal filters (ORBO32, Supelco Inc., Bellefonte, PA, USA) at 0.8 l/min for 24 hours at room temperature with supplemental light (16:8 L:D). An additional ORBO filter was used to monitor breakthrough. The chamber was washed with acetone and new ORBO filters were used for each new plant. The volatiles were eluted with 300 µl of methylene chloride (with 10 ng/µl of toluene as internal standard) and kept in a freezer (−20°C) and subjected to GC-MS analysis.

#### Chemical Analysis

We used a Shimadzu GCMS-QP5050A mass spectrometer (EI scan mode at 70 eV) coupled with a Shimadzu GC-17A equipped with a DB-1 capillary column (30 m ×0.25 mm ID, 0.25 µm film thickness; J&W Scientific, Folsom, California, USA) in splitless mode (1 min sampling) as previously described [23]. Helium was the carrier gas (1.0 ml/min flow rate), with the injector and interface temperature set at 280°C and 260°C. The oven temperature was programmed for 5 min at 40°C, and 15°C/min increase to 250°C and then held for 5 minutes. We identified the seven compounds by mass spectral matches to library spectra as well as by retention times matched to standards. The relative ratio (Table 1) was quantified based on ion abundances from GC-MS analyses according to the standard curves made from each authentic standard.

#### Synthetic blends—mimicking damaged shoots

Japanese beetle damage changed both the total amount and ratio of the seven compounds comprising the optimized blend. To decouple the effect of changes in ratio and concentration, we first manipulated the ratio of the “optimized blend” (Table 1) according to the volatile ratio released from damaged grape shoots and prepared a “damaged shoot blend” (JB damaged blend in Table 1). In addition, as the optimized blend had a ratio that was optimized previously in another flight tunnel study [23], and was slightly different than the one observed from the undamaged grape shoot (see Table 1 in [23]), we also prepared an “undamaged shoot blend” (JB undamaged blend in Table 1). The ratio reported in Table 1 was what we observed from the headspace of *V. riparia* shoots. To test the effect of concentration, we prepared all three different synthetic blends in Table 1 at two concentrations (10 times difference at 0.1 µg/µl and 1.0 µg/µl) for two doses (30 µg/septa and 300 µg/septa respectively).

#### Flight Tunnel Assays

Flight tunnel bioassay conditions were identical to the experiment 1. For this experiment, however, we used both grape shoots and synthetic blends as targets. For flights to grape shoots, a grape shoot covering a space of 15 cm H ×20 cm W was cut from the experimental grapevine (8 total) and immediately placed into a water pick just prior to testing. We used freshly cut shoots because there was no difference in upwind flight or landing between undamaged freshly cut shoots and potted grape plants [37] and we wanted to minimize target size and possible contamination from the pot and soil. For flights to synthetic blends, we used three horizontally placed rubber septa (10 cm apart) per flight to mimic the size of shoots used in flight tunnel. We loaded each septum with 300 µl of the synthetic blends prepared as described above at 0.1 µg/µl and 1.0 µg/µl.

#### Statistical analysis

The attractiveness of different synthetic blends (doubled ratio) and different grape shoots (control vs. Japanese beetle damage) to female *P. viteana* was analyzed using generalized linear models with the behaviors of leaving the cage, partial or full upwind flights, or landing as dependent variables and different mixtures as a fixed independent variable using binomial distribution with logit link function and maximum likelihood estimation (Proc Glimmix) [38]. The effect of Japanese beetle damage on the total amount and the ratio of seven key volatiles was analyzed using Proc Mixed [38] with Japanese beetle damage as fixed independent variable. The effect of the changed ratio (original blend vs. undamaged blend vs. damaged blend) and dose (30 µg/septa vs. 300 µg/septa) on female *P. viteana* behavior in the flight tunnel was also analyzed using generalized linear models with the behaviors of leaving the cage, partial or full upwind flights as dependent variables and different ratios and dose as fixed independent variables using binomial distribution with logit link function and maximum likelihood estimation (Proc Glimmix) [38].

## Results

### Experiment 1: Does doubling essential compound singly affect female *P. viteana* orientation behavior?

**Figure 1:**
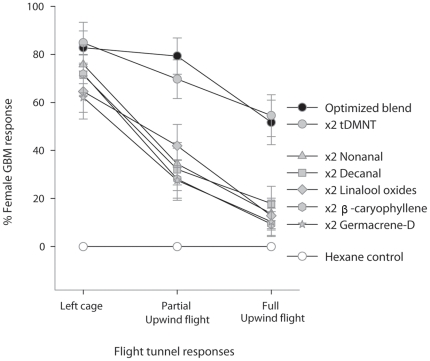
Response of female *P. viteana* to doubling the ratio of a single volatile constituent. Flight responses (% ±1 *SE*) of female *P. viteana* (*N* = 241) in the flight tunnel to hexane control, 7-component “optimized blend”, and six ratio-modified synthetic blends. Ratio modification was done systematically by doubling one compound in the optimized blend singly and is indicated by “x2” in the figure legend. Partial upwind flight was recorded when *P. viteana* made more than 50 cm of zig-zag upwind flight toward the target. Full upwind flight was the upwind flight to within 10 cm of the target. An average of 30 individual moths was flown to each target tested. tDMNT: 4,8-dimethyl-1,3(*E*),7-nonatriene.

With the exception of tDMNT, doubling any compound of the previously identified 7-component blend (the “optimized” blend) singly significantly reduced female full upwind flight by an average of 75.6% (*F*_6,18_ = 5.65, *P* = 0.002) and partial upwind flight by 58.6% (*F*_6,18_ = 4.81, *P* = 0.004) compared to the original blend (Fig. 1). Interestingly, modified blends, although less attractive, were still recognized by females, as doubling did not significantly affect the proportion of females that left the cage in the flight tunnel (*F*_6,18_ = 1.10, *P* = 0.39). Complete lack of female response to hexane control further supported female recognition of the modified blends.

### Experiment 2: Does Japanese beetle damage-induced modulation in ratio and concentration of key volatiles affect female *P. viteana* orientation behavior?

**Figure 2:**
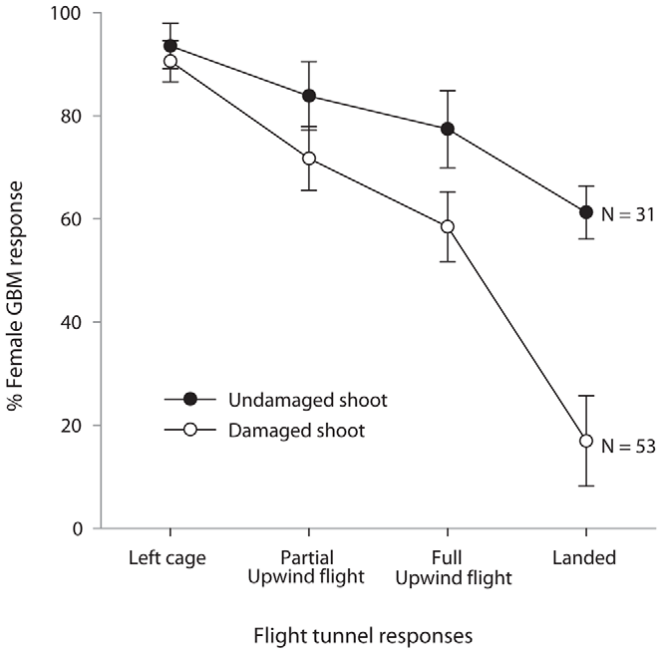
Effect of Japanese beetle foliar damage on female *P. viteana* flight response to host plant. Flight responses (% ±1 *SE*) of female *P. viteana* (*N* = 84) in the flight tunnel to Japanese beetle damaged vs. undamaged *V. riparia* shoots. Partial upwind flight was recorded when *P. viteana* made more than 50 cm of zig-zag upwind flight toward the target. Full upwind flight was the upwind flight to within 10 cm of the target.

Since the doubling of single compounds may not be representative of the variation observed in nature, during the summer of 2009 we modified host plant volatile profiles by inflicting Japanese beetle damage on clonal *V. riparia* grapevines. We tested damaged and undamaged grape shoots in the flight tunnel and found that Japanese beetle damage reduced close range female responses (*N* = 84)—landing by 72.3% (*F*_1,19_ = 15.75, *P*<0.001) and full upwind flight, marginally, by 24.5% (*F*_1,19_ = 3.10, *P* = 0.09) (Fig. 2). Japanese beetle damage was not associated with reduction in longer range female responses—partial upwind flight (*F*_1,19_ = 1.47, *P* = 0.24) or percent of females that left the cage (*F*_1,19_ = 0.30, *P* = 0.59).

**Figure 3:**
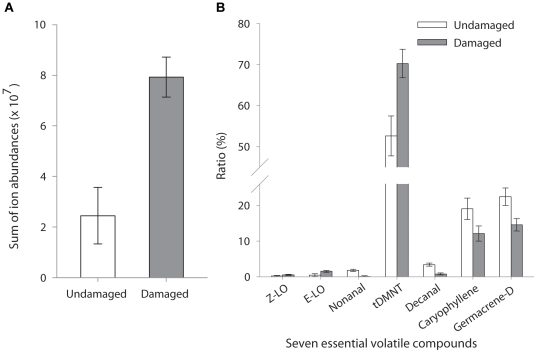
GC-MS analysis of headspace volatiles from Japanese beetle damaged vs undamaged grapevines. Modulation in the (A) concentration (as the sum of ion abundances from GC/MS analyses ± *SE*) and (b) ratio (% ±1 *SE*) of the 7 key volatile compounds emitted from Japanese beetle damaged vs. undamaged *V. riparia* shoots (*N* = 8). Z-LO: (*Z*)-linalool oxide; E-LO: (*E*)-linalool oxide; tDMNT: 4,8-dimethyl-1,3(*E*),7-nonatriene.

Reduced female response to damaged shoots was associated with more than a 2-fold damage-induced increase in total amount of the 7 key essential volatile compounds (*F*_1,7_ = 16.02, *P* = 0.005) (Fig. 3a) and/or modulations in the ratio of the 7 compounds (Fig. 3b). Although the total emission of 7 volatiles was significantly increased by beetle feeding, changes in the ratio among different volatile compounds were not uniform. In general, Japanese beetle damage increased the ratio of (*E*)- & (*Z*)-linalool oxide and tDMNT, but decreased the ratio of nonanal, decanal, β-caryophyllene and germacrene-D. More specifically, the damage induced a more than 2-fold relative increase in (*E*)-linalool oxide (*F*_1,7_ = 5.6, *P* = 0.05), whereas a 1.7-fold increase in (*Z*)-linalool oxide was not statistically significant (*F*_1,7_ = 2.15, *P* = 0.19). Damage also increased the ratio of tDMNT by 33.5% (*F*_1,7_ = 8.76, *P* = 0.02). Interestingly, the ratios of both aldehydes, nonanal and decanal, were significantly reduced by 95.1% (*F*_1,7_ = 19.79, *P* = 0.003) and 76.0% (*F*_1,7_ = 25.69, *P* = 0.001), respectively. Also, the ratio of germacrene-D was reduced by 34.9% in response to the Japanese beetle damage (*F*_1,7_ = 6.70, *P* = 0.04). β-caryophyllene showed a similar decrease (36.4%), although the decrease was only marginally significant (*F*_1,7_ = 3.56, *P* = 0.10).

**Figure 4:**
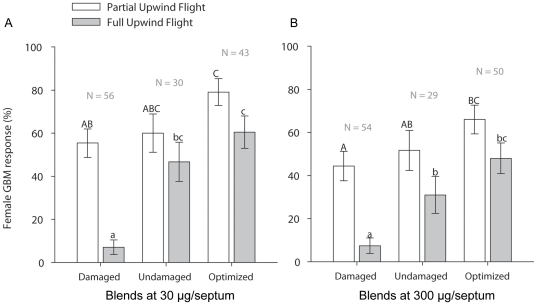
Response of female *P. viteana* to modulations in ratio and concentration. Partial and full upwind flight responses (% ±1 *SE*) of female *P. viteana* (*N* = 262) to three different synthetic blends (optimized blend vs. Japanese beetle undamaged blend vs. Japanese beetle damaged blend in Table 1) at two doses, (A) containing 30 µg of the 7 compounds per lure and (B) containing 300 µg of the 7 compounds per lure. Partial upwind flight was recorded when *P. viteana* made more than 50 cm of zig-zag upwind flight toward the target. Full upwind flight was the upwind flight to within 10 cm of the target. Different letters (capital letters for partial upwind flight response and small letters for full upwind flight response) on bars indicate significant differences (*P*<0.05).

To decouple the potential effects of ratio and concentration on the reduction of *P. viteana* response in flight tunnel, we tested females with three different synthetic blends (optimized, undamaged, and damaged blend; Table 1) at two doses (30 and 300 µg/septa). This test supported the hypothesis that the damage-induced decrease in female attraction was due to the changes in ratios of key volatiles (*F*_2,17_ = 20.62, *P*<0.001 for full upwind flight and *F*_2,17_ = 5.43, *P* = 0.02 for partial upwind flight) rather than overall increase in concentration (*F*_1,17_ = 1.26, *P* = 0.28 for full upwind flight and *F*_1,17_ = 3.17, *P* = 0.09 for partial upwind flight). No significant interactions between blend and dose were observed. The pattern of female response for full and partial upwind flights to different blends was similar (Fig. 4). Compared to the optimized and undamaged blends, female flights to the damaged blend showed a 88.3% and 84.8% (significant) reduction in full upwind flight at 30 µg/septa, and 84.6% and 76.1% reduction at 300 µg/septa. Similarly, females showed 30.0% and 32.7% reductions in partial upwind flight in response to the damaged blend compared to the optimized blend at 30 and 300 µg/septa level, respectively. However, we did not observe differences in partial upwind flights between the undamaged and damaged blends at either dose. Similar to the female response to shoots, there were no differences among different blends in proportion of females that left the cage.

## Discussion

Our results demonstrate that modulation in the ratios of essential compounds was sufficient to affect the host recognition behavior of female *P. viteana*. We confirmed this by using a previously identified 7-component optimized blend [23], and testing various ratio-modified (systematically doubling single compound) synthetic blends in the flight tunnel (Fig. 1) and also by mimicking increased and decreased ratios of the constituents in the optimized blend as emitted from grapevines damaged by one day of Japanese beetle feeding (Fig. 3 and 4). Although it is possible that one day of feeding may not be enough to elicit maximal inducible responses of volatiles from grape shoots as shown in some other systems [39] and the natural variation in emission of some plant volatiles, such as green leaf volatiles, can be much higher than the variation observed in this study [40], the changes in ratio of key volatile constituents, within the relatively narrow range tested in our study, resulted in up to 87% reduction in female full upwind flight in the flight tunnel. This supports the hypothesis that the specificity of a ubiquitous volatile blend to this specialist herbivore is determined, at least in part, by the ratio of key volatile compounds. On the other hand, the fact that doubling tDMNT did not affect *P. viteana* female response indicates that *P. viteana* has the plasticity or the capacity to orient to an odor source despite some variation in the ratio as well as the concentration of the overall mixture. It is possible that a greater increase of tDMNT than we tested, such as 10-times or 100-times increase, could negatively affect *P. viteana* female behavior and could be used by females to eavesdrop on host plant quality, although a recent study that reports no effect of 100-times increase in benzonitrile (one of the bioactive peach shoot volatiles for *Cydia molesta*, oriental fruit moth) on female *C. molesta* attraction is consistent with our results [34].

The limited evidence for the importance of the ratio of essential volatile compounds in herbivore host location behavior, and also in pollinator-flower and natural enemy-herbivore-plant interaction, is mostly consistent with our findings [8], [28], [29], [34], [41], [42]. This indicates that a diversion from a naturally produced ratio of host plant volatiles or a ratio specific to preferred host genotypes/cultivars often results in a decreased behavioral response of insects. Results from our study involving explicit ratio manipulation of the essential volatile compounds confirmed this by showing that not only artificial ratio changes (doubling single compound) but also naturally-caused increase and decrease in the ratio of host volatile compounds could affect specificity in the olfactory-based host processing in this phytophagous insect and possibly other species.

Although changes in volatile ratios significantly affected female behavior, these changes did not completely eliminate female *P. viteana* upwind orientation flights. Instead, changes in ratio affected female orientation response at close range to the host plant rather than upwind oriented flight from longer range. At least within the range of tested ratios, we found that 50% of the females could recognize ratio-modified blends and still fly half way toward the target (partial upwind flight) but only 6% went the entire distance. This compares with about the same or only a slightly greater level of partial upwind flight to the “correct” ratio, but 50% of females went the entire distance (Fig. 4). We also found a similar outcome using actual Japanese beetle damaged and undamaged shoots (Fig. 2). Together these results suggest that, although the ratio of some host volatile compounds plays a critical role in the fine-tuning of host recognition, *P. viteana* may still be able to perceive and interpret “off-ratio” blends as the correct host, and approach to the vicinity of the plant, resulting in increased probability of host recognition. This plasticity in the perception of variable ratios is to be expected since the plants are not tightly regulating the production and release of volatile compounds for the benefit of the herbivore.

In our previous study we demonstrated plasticity, or redundancy, in the attractive blend for *P. viteana*, evidenced by maximal response levels to two mixtures that differed slightly in volatile composition [23]. In the current study, we found plasticity in *P. viteana*'s ability not only to evaluate potential host quality by interpreting different ratios, but also to generalize different ratios of a blend with the same composition. For example, although there were significant differences in ratios between the undamaged and optimized blend (Table 1), we did not observe differences in orientation behavior (Fig. 4). This suggests that even with the same composition there is more than one optimum ratio that *P. viteana* can recognize. This plasticity, or redundancy, appears to be a natural response of phytophagous insects considering the large variation in plant volatile emission reported in the literature [10].

Female *P. viteana* were sensitive to volatile compounds produced from more than one biosynthetic pathway. It has been demonstrated that the ratio of volatile compounds belonging to the same biosynthetic pathway or similar functional groups changed more consistently under various conditions than compounds that are more distinct [11], [12], [41]. In our experiment, Japanese beetle damage reduced the ratios of both aldehydes (nonanal and decanal) and both sesquiterpenes (caryophyllene and germacrene-D) while it induced higher production of both isomers of linalool oxides and tDMNT. This type of constraint on change in ratio within a biosynthetic pathway, coupled with differences in ratios between compounds from distinct biosynthetic pathways, might provide enhanced information content. In other words, the activity and thus the amounts of volatile compounds produced by different biosynthetic pathways could co-vary differently depending on the condition of host plant or by different plant species and, thus, provide a biosynthetic pathway-based hint on host plant identity to foraging herbivores [12]. This may partially explain the discrepancy of why the ×2 increase of tDMNT in Experiment 1 did not reduce the female flight response, while 33.5% increase of tDMNT combined with a decrease in aldehydes and sesquiterpenes and increase in linalool oxides did reduce the female flight response.

The evolutionary significance of the avoidance response of *P. viteana* females to Japanese beetle damaged grapevines is unclear. Japanese beetle has only been in the Northeast for about 60 years and did not evolve with *P. viteana*, a native to the eastern USA. Although some insects, such as Japanese beetle, have been shown to be attracted to plants damaged by conspecifics or different insect species [43], the diminished attraction to damaged plants that we observed for *P. viteana* could, however, be a more general response of herbivores to reduced host quality [21] either with respect to decreased nutritional value/increased defense [44] and/or as increased risk from natural enemies [16], [42], [45], [46]. Or it could be that these damage-induced changes in the odor profile temporarily made the host unrecognizable for *P. viteana*. The fitness consequences of avoiding damaged foliage based on volatile signals needs to be further investigated as well as the time course of induced changes in attraction.

In addition to changes in ratio and concentration in response to feeding by Japanese beetle, GC-EAD and GC-Mass spec analysis of emissions also indicated the induction of some novel, antenally active compounds (unpublished data, D.H. Cha). Some of these could also have contributed to decreases in female orientation to shoots in the flight tunnel. However, simply mimicking the increase and decrease in ratio of the seven essential compounds was sufficient to achieve the same level of reduction in orientation as observed for damaged shoots, indicating induced compounds may not be the principal factor in this system.

To date, the majority of studies investigating plant volatile mediated plant-insect interactions have focused on the role of herbivore-induced plant volatile compounds (i.e., novel induction or large increase in the amount) in the context of plant defense [19], [22], although herbivore damage may result in both increased and decreased emission of volatiles from certain biosynthetic pathways [47] as observed in our study. Herbivorous insects have been very successful on earth, in part, due to their ability to eavesdrop on the host plant signal—to locate the correct species in a favorable condition and avoid unfavorable host conditions in mid-air, even before contact. The results reported here indicate that herbivores have the potential to determine host plant conditions by detecting the changes in volatile emissions of not only the novel or highly induced compounds [21], but also through the detection of small modulations in ratios of constitutively released volatile compounds that they use for host recognition. If herbivores can generally avoid an unfavorable host, this interaction has the potential to alter the outcome of the direct and indirect plant defenses against herbivores.
